# Socioeconomic Determinants of Cognitive Aging: A Cross-Country Comparison Between England and China

**DOI:** 10.1093/geroni/igab046.463

**Published:** 2021-12-17

**Authors:** Dorina Cadar, Yaohui Zhao, Li Yan, Laura Brocklebank, Andrew Steptoe

**Affiliations:** 1 University College London, London, England, United Kingdom; 2 Peking University, Guangzhou, Guangdong, China (People's Republic)

## Abstract

Lower educational attainment is associated with a higher risk of dementia and a steeper cognitive decline in older adults. However, less clear is how other socioeconomic markers contribute to cognitive ageing and if these socioeconomic influences on cognitive ageing differ between England and China. We examined the relationship of education, household wealth, and urbanicity with cognitive performance and rate of change over 7-8 years follow up in the English Longitudinal Study of Ageing and Chinese Health and Retirement Longitudinal Study, national representative samples of England and China. We found that the rate of cognitive change appears to be socioeconomically patterned, primarily by education and area-based characteristics (urban vs rural), with a stronger impact of inequalities seen in rural China. Public health strategies for preventing cognitive decline and dementia should target socioeconomic gaps to reduce health disparities and protect those particularly disadvantaged in England and China.

